# Costs and clinical benefits of enhanced recovery after surgery (ERAS) in pancreaticoduodenectomy: an updated systematic review and meta-analysis

**DOI:** 10.1007/s00432-022-04508-x

**Published:** 2023-01-11

**Authors:** Lyrics Noba, Sheila Rodgers, Lawrence Doi, Colin Chandler, Deepak Hariharan, Vincent Yip

**Affiliations:** 1 School of Health in Social Science, University of Edinburgh, 24 Buccleuch Place, Edinburgh, EH8 8LN UK; 2 Hepato-Pancreato-Biliary (HPB) Unit, Royal London Hospital (Barts Health NHS Trust), London, E1 1FR UK

**Keywords:** Enhanced recovery after surgery, Pancreaticoduodenectomy, Systematic review, Meta-analysis

## Abstract

**Purpose:**

ERAS is a holistic and multidisciplinary pathway that incorporates various evidence-based interventions to accelerate recovery and improve clinical outcomes. However, evidence on cost benefit of ERAS in pancreaticoduodenectomy remains scarce. This review aimed to investigate cost benefit, compliance, and clinical benefits of ERAS in pancreaticoduodenectomy.

**Methods:**

A comprehensive literature search was conducted on Medline, Embase, PubMed, CINAHL and the Cochrane library to identify studies conducted between 2000 and 2021, comparing effect of ERAS programmes and traditional care on hospital cost, length of stay (LOS), complications, delayed gastric emptying (DGE), readmission, reoperation, mortality, and compliance.

**Results:**

The search yielded 3 RCTs and 28 cohort studies. Hospital costs were significantly reduced in the ERAS group (SMD = − 1.41; CL, − 2.05 to − 0.77; *P* < 0.00001). LOS was shortened by 3.15 days (MD = − 3.15; CI, − 3.94 to − 2.36; *P* < 0.00001) in the ERAS group. Fewer patients in the ERAS group had complications (RR = 0.83; CI, 0.76–0.91; *P* < 0.0001). Incidences of DGE significantly decreased in the ERAS group (RR = 0.72; CI, 0.55–0.94; *P* = 0.01). The number of deaths was fewer in the ERAS group (RR = 0.76; CI, 0.58–1.00; *P* = 0.05).

**Conclusion:**

This review demonstrated that ERAS is safe and feasible in pancreaticoduodenectomy, improves clinical outcome such as LOS, complications, DGE and mortality rates, without changing readmissions and reoperations, while delivering significant cost savings. Higher compliance is associated with better clinical outcomes, especially LOS and complications.

## Introduction

In 1997, (Kehlet May 1997) introduced a multimodal approach to manage postoperative complications, which later evolved into enhanced recovery after surgery (ERAS). ERAS is a holistic and multidisciplinary pathway that incorporate various evidence-based interventions to accelerate recovery and reduce length of stay (LOS). Furthermore, it aimed to standardise care for patients undergoing specific procedures, with a view to improving clinical outcomes. ERAS was initially implemented in colorectal surgery. Due to its success, it was quickly adopted in other surgical specialities.

Pancreatic surgery is traditionally associated with high mortality and complication rates. Few decades ago, mortality in pancreatic surgery was as high as 25%, but has now fallen to under 5% owing to recent advances in diagnosis, surgical techniques and improvement in perioperative care management (Gooiker et al. 2014). However, complications tend to remain very high, ranging between 40 and 60% (Lermite, et al. 2013; Kunstman et al. 2019). Complications such as postoperative pancreatic fistula and delayed gastric emptying (DGE) are identified as the primary causes of delayed recovery which often require further radiological or surgical interventions (Zhang et al. 2020).

The past decade has seen various ERAS guidelines published for multiple surgical specialties including colorectal, cardiac, orthopaedic, breast and gastrointestinal surgery. The first ERAS guidelines for pancreatoduodenectomy were published by the ERAS society in 2012 (Lassen et al. 2012). The updated guidelines published in 2020 contain 27 elements, covering the three phases of perioperative care (preoperative, intra-operative and postoperative), including preoperative education, minimally invasive techniques, pain control and early mobilisation and feeding (Melloul et al. 2019).

The impact of ERAS has been widely studied in various surgical specialities including upper gastrointestinal surgeries with good results. In recent years, many studies have been published on the effect of ERAS in pancreatic surgery. These studies have demonstrated that implementation of the ERAS pathway in pancreatic surgery is safe and reduces LOS and complications without increasing mortality rates and readmissions. However, evidence on cost benefit of ERAS programmes in pancreatic surgery remains scarce. A recent meta-analysis of 27 studies demonstrated significant cost savings following the implementation of the ERAS pathway in liver surgery (Noba et al. 2020). To date, no meta-analysis has been conducted to evaluate the impact of ERAS in pancreaticoduodenectomy on hospital costs. The aim of this review is to investigate cost benefit, compliance and clinical benefits of ERAS in pancreaticoduodenectomy.

## Methods

### Search strategy

This review was conducted in compliance with PRISMA (preferred reporting items for systematic reviews and meta-analyses) guidelines for systematic reviews and meta-analysis (Moher 2010). Multiple databases, (Medline, Embase, PubMed, CINAHL, and the Cochrane library), were searched to identify studies published between January 2000 and December 2021. The search was restricted to English language publications. A further search was conducted on the reference lists of relevant eligible studies and Systematic Reviews. The search terms such as ‘ERAS’, ‘FTS’, ‘Fast track’, ‘Enhanced recovery’, ‘Clinical pathway’, ‘Critical pathway’, ‘Accelerated recovery surgery’, ‘Pancreas’, ‘Pancreatic’, ‘Whipple’, ‘Pancreatectomy’, ‘Pancreatoduodenectomy’, ‘Pancreaticoduodenectomy’ were applied using Boolean operators (OR and AND).

### Inclusion/exclusion criteria

Studies were eligible for inclusion if they met all of the following criteria (1) adult patients undergoing pancreaticoduodenectomy (2) compared ERAS to traditional care (3) reported at least one of the following outcomes: Hospital Costs, LOS, Complications, Compliance, Delayed Gastric Emptying (DGE), Mortality rates, Readmissions and Reoperations. Studies were excluded if they were non-elective or transplant patients, non-pancreaticoduodenectomy (PD), non-English and not comparing ERAS to traditional care.

### Data extraction

Eligible studies and relevant data were retrieved and extracted by the first author. Data were extracted using a data extraction sheet agreed by all authors and were subsequently validated by other authors. Data extracted included; authors’ names, year of publication, study design, patient’ characteristics (ASA grade, age, sex and BMI), type of surgery, surgical techniques, outcomes measured, sample size, follow-up period and ERAS items.

### Outcomes of interest

The primary outcomes for this systematic review were hospital costs. Secondary outcomes included: length of stay, compliance, complications, DGE, mortality, readmission and reoperation. LOS is defined by the total number of days a patient spent in the hospital prior to discharge.

### Quality assessment

**Fig. 1:**
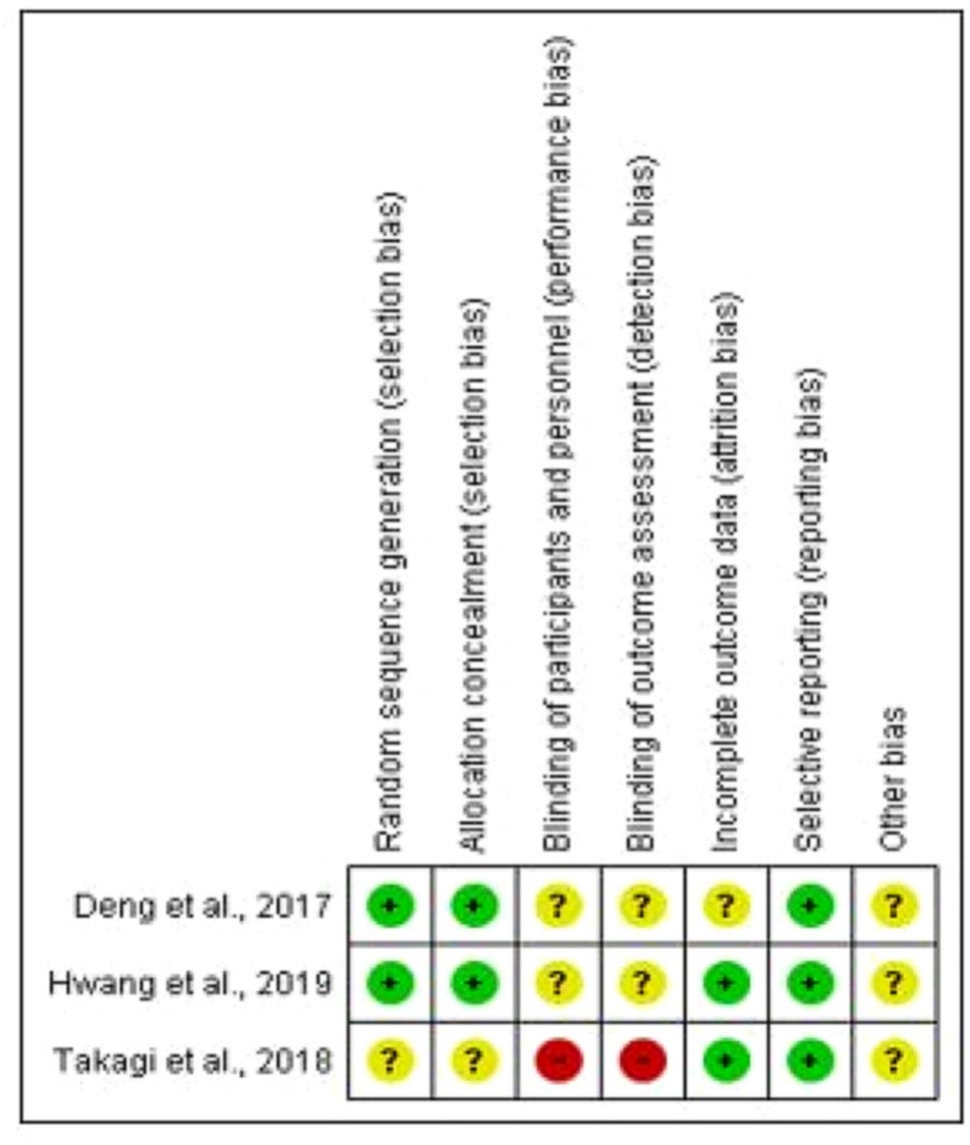
Summary of risk of bias of randomised control trials

In line with the Cochrane Collaboration’s risk of bias tool, the quality of the Randomised Control Trials (RCTs) were assessed against the following domains: random sequence generation, allocation concealment, blinding of participants and personnel, blinding of outcome assessment, incomplete outcome data and selective reporting (Higgins et al. 2011). See Fig. 1 for summary of risk of bias of RCTs. The methodological quality of the cohort studies were assessed using the Newcastle–Ottawa Quality Assessment Scale (NOS) (Hartling et al. 2012). The NOS has a maximum of 9 stars (Selection 4 stars, Comparability 2 stars and Exposure 3 stars).

### Statistical analysis

This review was conducted using Review Manager (RevMan) version 5.4 (Collaboration 2020). Risk ratio was used for all dichotomous variables, weight mean difference or weight standardised mean difference for continuous variables with 95% confidence interval (CI). Statistical significance level was set at *p* < 0.05. Statistical heterogeneity was assessed using a chi-squared test (*χ*^2^), *I*^2^ statistic. A *P* < 0.1 was considered to be a statistically significant heterogeneity. A fixed effect model was applied for pooling. Where there is substantial evidence of heterogeneity (*I*^2^ > 60%), a random effect model was applied instead. Using the method recommended by (Hozo et al. 2005), study data presented as medians and interquartile ranges were converted to mean and standard deviation (SD). Standard deviation from a study with similar sample size was used with the mean as suggested by (Furukawa et al. 2006). The presence of publication bias was assessed using Funnel plots.

## Results

### Search results

**Fig. 2:**
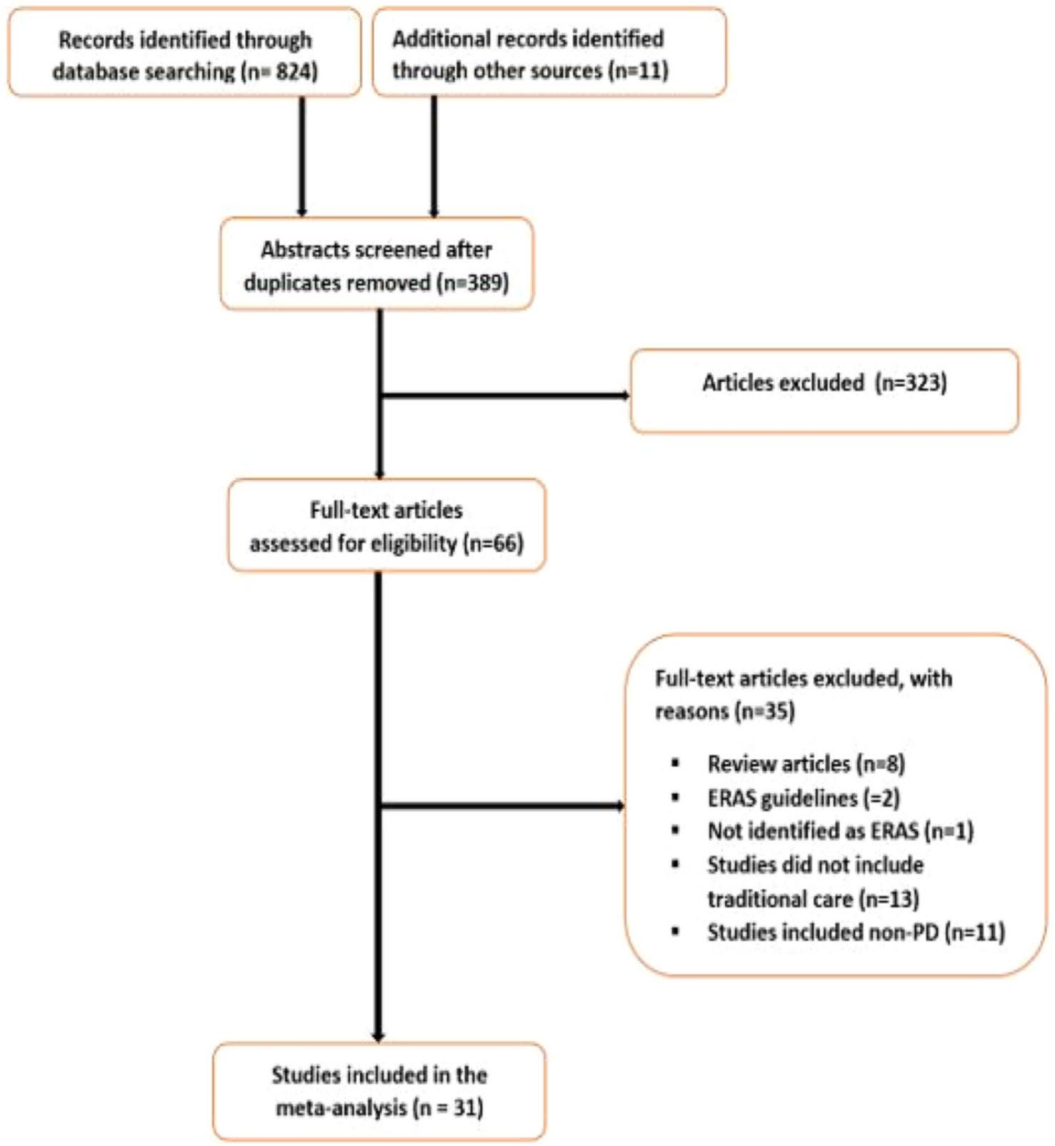
Flow chart of study selection process

An initial search resulted in 835 studies. After inclusion/exclusion criteria were applied, 31 final studies were included in the meta-analysis. See Fig. 2 for the PRISMA flow chart.

### Characteristics of included studies

A total of 5382 patients were included in this review (range between 41 and 635, per study), with 2776 patients in the ERAS group and 2606 patients in the traditional care group. Full details of the characteristics for included studies is shown in Table 1.

**Table 1:** Characteristics of included studies

Studies	Study design	Type of surgery	Surgery approach	ASA Grade		Age		Sex		BMI		Sample size		†NOS (9*)
				Pre-ERAS	ERAS	Pre-ERAS	ERAS	Pre-ERAS	ERAS	Pre-ERAS	ERAS	Pre-ERAS	ERAS	
				I/II/III/IV	I/II/III/IV			M/F	M/F					
Balzano et al. (2008)	Cohort study	PD/PPPD	Ns	Ns	Ns	62·9 (26–84)	64·3 (33–88)	148/104	155/97	Ns	Ns	252	252	6
Braga et al. (2014)	Cohort study	PD/PPPD	Open	4/82/29/0/0	4/88/23/0/0	69 (61–74)	69 (61–74)	66/49	66/49	23.1 (21–25)	23.7 (21–25)	115	115	7
Coolsen et al. (2014)	Cohort study	PPPD/PD	Ns	NS	Ns	62 ± 13	67 ± 11†	58/39	44/42	Ns	Ns	97	86	5
Dai et al. (2017)	Cohort study	PPPD/PD	Ns	15/75/8/0/0	18/44/6/0/0	59.2 (14–83)	58.5 (18–69)	51/47	34/34	22.94 (14.95–34.64)	21.48 (18.55–28.40)	98	68	5
Deng et al. (2017)	RCT	PPPD/PD	Ns	Ns	Ns	51.3 ± 15.0	54.5 ± 12.7	46/37	46/30	I–II (64)/III–IV (19)	I–II (54)/III–IV (22)	83	76	
French et al. (2009)	Cohort study	PD/PPPD	Ns	Ns	Ns	66.2 (10.3)	53.8 (11.6)	Ns	Ns	Ns	Ns	49	9	5
Hilal et al. (2013)	Cohort study	PD	Open	6/15/3/0/0	4/15/1/0/0	70 (61–76)	68.5 (65–72)	10/14	10/10	Ns	Ns	24	20	5
Hwang et al. (2019)	RCT	PD/PPPD/SSPD	Open	18/100/6/0/0	9/99/15/0/0	62.9 + 9.2	63.3 + 9.2	81/43	72/51	24.2 + 3.0	24.3 + 3.1	124	123	
Joliat et al. (2015)	Cohort study	PPPD/PD	Ns	I/II (67)/20/0/0	I/II (50)/24/0/0	67 (55–75)	67⋅5 (57–74)	56/31	39/35	24⋅2 (22⋅1–27⋅3)	23⋅9 (22⋅1–26⋅7)	87	74	5
Kagedan et al. (2017)	Cohort study	PD	Open	Ns	Ns	65.5 (58–74)	65 (56–74)	31/43	74/47	Ns	Ns	74	121	5
Kennedy et al. (2007)	Cohort study	PD	Ns	C	Ns	61.3 ± 2.0	63.9 ± 1.3	23/21	41/50	Ns	Ns	44	91	5
Kobayashi et al. (2014)	Cohort study	PD/PPPD/SSPD	Ns	Ns	Ns	65.4 ± 10.8	67.5 ± 10.7	62/28	61/39	25.0 ± 4.54	21.6 ± 3.54	90	100	5
Kowalsky et al. Jun. (2019)	Cohort study	PD	Robotic/Open	0/12/106/5	0/14/103/14	66.3 ± 10.4	68.2 ± 9.8	70/61	64/59	28.2 ± 6.3	26.8 ± 5.9	131	123	6
Morales Soriano, et al. (2015)	Cohort study	PD	Ns	Ns	I (21)/II–III (20)	66.7 (41–84)	61.3 (44–80)	27/17	24/17	Ns	Ns	44	41	7
Nikfarjam et al. Jan. (2013)	Cohort study	PPPD/PD	Ns	Ns	0/5/15	62 (15–81)	68 (45–81)	12/9	13/7	24 (19–34)	25 (19–42)	21	20	5
Nussbaum et al. (2015)	Cohort study	PD	Open/Lap	Ns	Ns	62.1 + 11.5	65.5 + 10.1	67/75	39/61	27.1 + 6.5	26.2 + 4.6	142	100	6
Partelli et al. (2016)	Cohort study	PPPD	Open	5/42/1/0/0	2/13/7/0/0	77.5 (75–82)	77 (75–82)	33/33	14/8	25 (18–32)	25 (21–31)	66	22	5
Ahanatha Pillai et al. (2014)	Cohort study	PD	Ns	Ns	Ns	47.6 ± 12.0	44.2 ± 15.9	10/10	11/9	Ns	Ns	20	20	7
Shah et al. (2016)	Cohort study	PPPD/PD	Ns	7/27/12/2/0	18/79/36/9/0	59.1 ± 10.4	61.9 ± 9.1	30/16	84/58	21.5 ± 2.7	21.5 ± 2.5	46	142	5
Shao et al. (2015)	Cohort study	PPPD/PD	Ns	Ns	Ns	57.05 ± 12.30	56.96 ± 11.50	184/126	194/131	Ns	Ns	310	325	7
Su, et al. (2017)	Cohort study	PD	Ns	> II (5)	> II (5)	61 ± 11	62 ± 9	18/13	19/12	22.7 ± 2.8	22.4 ± 3.0	31	31	9
Sutcliffe et al. (2015)	Cohort study	PPPD/PD	Ns	Ns	Ns	66 (35–83)	67 (18–83)	37/28	40/25	25.4 ± 4.4	27.3 ± 5.8	65	65	5
Takagi et al. (2019)	RCT	PPPD/SSPPD/PD	Ns	6/26/5/0/0	3/23/11	66.8 (9.3)	67.8 (9.7)	20/17	20/17	21.7 (2.8)	22.1 (3.0)	37	37	
Téoule et al. (2020)	Cohort study	PPPD/PD	Ns	6/39/27/3/ (72)	11/71/65/0 (1)	64.2	65.6	87/60	90/58	25.7	25.6	147	148	5
Tremblay St-Germain, et al. (2017)	Cohort study	PPPD/PD	Ns	0/15/55/4/0	0/17/49/17/0	66 (24–84)	65 (29–85)	31/43	44/39	25 (15–36)	26 (16–45)	74	83	5
Kolk et al. (2017)	Cohort study	PD	Ns	Ns	Ns	66 (58–72)	66 (57–72)	35/17	56/39	Ns	Ns	52	95	5
Vanounou et al. (2007)	Cohort study	PPPD/PD	Ns	1/33/30/0/0	2/53/84/6/0	64	64	Ns	Ns	Ns	Ns	64	145	5
Williamsson et al. (2015)	Cohort study	Ns	Ns	6/27/17/0/0	2/28/20/0/0	67 (25–81)	69 (15–80)	26/24	31/19	25⋅2 (16⋅3–33⋅4)	24⋅3 (19⋅4–36⋅2)	50	50	7
Williamsson et al. (2019)	Cohort study	PD	Ns	Ns	Ns	Ns	NS	Ns	Ns	Ns	Ns	50	55	5
Zhu et al. (2020)	Cohort study	PD	Ns	21/33/15/0/0	17/34/13	64.1 ± 11.5	64.3 ± 7.9	32/37	27/37	42	44	69	64	7
Zouros et al. (2016)	Cohort study	PPPD/PD	Ns	18/27/5/0/0	26/33/16/0/0	63.9 ± 11.6	65.9 ± 10.5	34/16	46/29	Ns	Ns	50	75	8

*PD* pancreatoduodenectomy, *SSPD* subtotal stomach-preserving pancreaticoduodenectomy, *PPPD*  pylorus preserving pancreaticoduodenectomy, *SSPPD* subtotal stomach preserving pancreaticoduodenectomy, *Ns* not stated

^†^Newcastle–Ottawa quality assessment scale (maximum 9 stars)

The number of ERAS items applied across the studies varied substantially. While, five studies did not provide lists of items utilised in their study (French et al. 2009), (Téoule et al. 2020). A detailed list of ERAS items utilised by individual studies is shown in Table 2. Three studies were RCTs (Deng et al. 2017), (Takagi et al. 2019), while the remaining studies were cohort studies (French et al. 2009), (Téoule et al. 2020; Joliat et al. 2015), (Hilal et al. 2013). The surgical approach was reported in six studies. Of these studies, four were open surgery (Hwang et al. 2019; Partelli et al. 2016; Braga et al. 2014; Hilal et al. 2013), one combined robotic and open surgery (Kowalsky et al. 2019), while the remaining study utilised a combination of open and laparoscopic approach (Nussbaum et al. 2015). Full details of characteristics for included studies is shown in Table 1.

**Table 2:** Summary of ERAS items

ERAS items	Balzano et al. (2008)	Braga et al. (2014)	Coolsen et al. (2014)	Dai et al. (2017)	Deng et al. (2017)	Hilal et al. (2013)	Hwang et al. (2019)	Joliat et al. (2015)	Kagedan et al. (2017)	Kennedy et al. (2007)	Kobayashi et al. (2014)	Kowalsky et al. Jun. (2019)	Morales Soriano, et al. (2015)
Preoperative counselling	Ns	+	+	+	+	+	+	+	Ns	+	+	Ns	Ns
Prehabilitation	Ns	Ns	Ns	Ns	Ns	Ns	Ns	Ns	Ns	Ns	Ns	Ns	Ns
Avoid Preoperative biliary drainage	Ns	–	Ns	–	Ns	Ns	+	Ns	Ns	Ns	–	Ns	–
Preoperative smoking and alcohol cessation	Ns	Ns	Ns	Ns	Ns	Ns	+	Ns	Ns	Ns	Ns	+	Ns
Preoperative nutrition	Ns	Ns	Ns	Ns	Ns	Ns	Ns	Ns	Ns	Ns	Ns	+	Ns
Avoid Perioperative oral immunonutrition	Ns	–	Ns	Ns	Ns	Ns	–	Ns	Ns	Ns	Ns	Ns	Ns
Preoperative fasting and treatment with carbohydrates	Ns	Ns	+	+	Ns	+	+	+	Ns	Ns	Ns	+	Ns
Pre-anaesthetic medication	Ns	–	–	Ns	Ns	Ns	+	Ns	Ns	Ns	Ns	Ns	Ns
Anti-thrombotic prophylaxis	Ns	Ns	+	Ns	Ns	+	+	+	Ns	+	–	Ns	Ns
Antimicrobial prophylaxis and skin preparation	Ns	Ns	+	+	Ns	Ns	+	Ns	Ns	Ns	Ns	Ns	+
Epidural analgesia	+	+	+	+	Ns	–	+	+	+	–	Ns	Ns	+
Postoperative intravenous and per oral analgesia	Ns	+ POD4	+ POD4	Ns	Ns	+ POD4	Ns	Ns	+ POD2	+ POD4	Ns	Ns	+ POD4
Wound catheter and transversus abdominis plane (TAP) block	Ns	Ns	Ns	Ns	Ns	Ns	Ns	Ns	Ns	Ns	Ns	Ns	Ns
Postoperative nausea and vomiting (PONV) prophylaxis	–	+	+	Ns	+	Ns	+	+	Ns	Ns	Ns	Ns	+
Avoiding hypothermia	Ns	+	+	+	+	+	+	+	Ns	Ns	Ns	Ns	+
Postoperative glycaemic control	Ns	Ns	Ns	Ns	Ns	Ns	+	+	+	Ns	Ns	Ns	Ns
Avoid Nasogastric intubation	–	–	+	–	–	–	+	+	Ns	–	–	Ns	–
Fluid balance	Ns	+	+	+	+	+	+	+	Ns	+	Ns	+	Ns
Early removal of Perianastomotic drainage	Ns	Not used	+ POD4	+ POD3	+ POD7 -10	+ POD4	+ POD3	+ POD 3/4	+ POD3	+ POD3	+ POPOD5	+ POD3–5	+ POPOD4
Avoid Somatostatin analogues	Ns	–	+	Ns	–	–	+	+	Ns	Ns	–	–	–
Removal of Urinary drainage	Ns	Ns	+ POD2	+ POD1	+ POD3	+ POD3	+	+ POD3	Ns	+ POD2	Ns	Ns	+ POD3
Prevention of DGE	Ns	Ns	Ns	Ns	Ns	Ns	+	Ns	Ns	Ns	Ns	Ns	Ns
Stimulation of bowel movement	Ns	Ns	Ns	Ns	Ns	Ns	+ Chew gum	Ns	Ns	Ns	Ns	Ns	Ns
Postoperative artificial nutrition	Ns	+	Ns	Ns	Ns	Ns	–	Ns	Ns	Ns	+	Ns	+
Early and scheduled mobilization	+ POD1	+ POD1	+ POD1	+ POD1	+ POD1/2	+	+	+ POD0	+ POD1	+ POD1	ns	+	+ POD4
Minimal invasive surgery	Ns	Ns	Ns	Ns	Ns	Ns	Ns	Ns	Ns	Ns	Ns	+	Ns
Systemic audit	Ns	Ns	Ns	Ns	Ns	Ns	+	+	Ns	Ns	Ns	Ns	Ns

ERAS items	Nussbaum et al. (2015)	Partelli et al. (2016)	Ahanatha Pillai et al. (2014)	Shah et al. (2016)	Su et al. (2017)	Sutcliffe et al. (2015)	Takagi et al. (2019)	Tremblay St-Germain, et al. (2017)	Kolk et al. (2017)	Vanounou et al. (2007)	Williamsson et al. (2019)	Zhu et a. (2020)	Zouros et al. (2016)
Preoperative counselling	Ns	+	+	+	+	+	+	+	+	+	+	+	+
Prehabilitation	Ns	Ns	Ns	Ns	Ns	Ns	Ns	Ns	Ns	Ns	Ns	Ns	Ns
Avoid Preoperative biliary drainage	Ns	–	Ns	Ns	Ns	Ns	–	–	Ns	Ns	Ns	Used	–
Preoperative smoking and alcohol cessation	Ns	Ns	Ns	Ns	Ns	Ns	Ns	Ns	Ns	Ns	Ns	Ns	Ns
Preoperative nutrition	Ns	Ns	Ns	Ns	Ns	Ns	Ns	Ns	+	Ns	Ns	–	Ns
Avoid Perioperative oral immunonutrition	Ns	Ns	Ns	Ns	Ns	Ns	–	Ns	+	Ns	Ns	Ns	Ns
Preoperative fasting and treatment with carbohydrates	Ns	Ns	Ns	Ns	–	+	+	Ns	Ns	Ns	+	Ns	+
Pre-anaesthetic medication	Ns	Ns	Ns	Ns	Ns	Ns	Ns	Ns	Ns	–	Ns	Ns	–
Anti-thrombotic prophylaxis	Ns	+	Ns	Ns	Ns	+	+	+	+	+	+	Ns	Ns
Antimicrobial prophylaxis and skin preparation	Ns	+	Ns	Ns	+	Ns	Ns	Ns	+	+	+	Ns	Ns
Epidural analgesia	+	+	+	Ns	+	+	+	+	+	Ns	+	+	+
Postoperative intravenous and per oral analgesia	+ POD4/5	+ POD3	Ns	+ POD 4	+ POD 3	Ns	Ns	+ POD2	Ns	Ns	Ns	Ns	+ POD3
Wound catheter and transversus abdominis plane (TAP) block	Ns	Ns	Ns	Ns	Ns	Ns	Ns	+ D3	Ns	Ns	Ns	Ns	Ns
Postoperative nausea and vomiting (PONV) prophylaxis	Ns	Ns	+	Ns	+	+	Ns	Ns	+	Ns	+	Ns	Ns
Avoiding hypothermia	Ns	Yes	Ns	Ns	Ns	+	+	Ns	Ns	Ns	Ns	+	+
Postoperative glycaemic control	Ns	+	+	Ns	Ns	Ns	+	+	+	Ns	Ns	Ns	Ns
Avoid Nasogastric intubation	Ns	–	–	–	Ns	–	+	–	–	–	–	–	–
Fluid balance	Ns	+	+	Ns	+	Ns	+	Ns	+	Ns	+	+	+
Early removal of Perianastomotic drainage	+	+ POD3	+ POD3	+ POD5/6	Ns	+ POD3	+	+ POD3	+	+	+ POD 3	+ D4–5	+ POD5
Avoid Somatostatin analogues	–	+	Ns	–	Ns	–	Ns	–	Ns	Ns	Ns	Ns	–
Removal of Urinary drainage	+ POD2	+ POD1	+ POD2	+ POD1	+ POD 2	+ POD1/2	+ POD2/3	+ POD3	Ns	_+_	+ POD 6	+ D1	+ POD2
Prevention of DGE	Ns	Ns	Ns	Ns	Ns	Ns	Ns	Ns	Ns	Ns	Ns	Ns	Ns
Stimulation of bowel movement	Ns	Ns	Ns	Ns	Ns	Ns	Ns	Ns	Ns	Ns	Ns	Ns	+ Chewing gum
Postoperative artificial nutrition	Ns	Ns	Ns	Ns	+	Ns	Ns	Ns	Ns	Ns	Ns	Ns	+
Early and scheduled mobilization	+ POD2	+ POD1	+ POD1	+ POD2	+ POD 1	+	+	+ POD1	+ POD1	ns	+	ns	+ POD1
Minimal invasive surgery	+	–	Ns	Ns	–	Ns	Ns	Ns	Ns	–	Ns	Ns	Ns
Systemic audit	Ns	Ns	Ns	Ns	Ns	Ns	Ns	Ns	Ns	Ns	Ns	Ns	Ns

*Ns* not stated, *POD* postoperative day

+ Policy applied

–Policy not applied

### Sensitivity analysis and publication bias

**Fig. 3:**
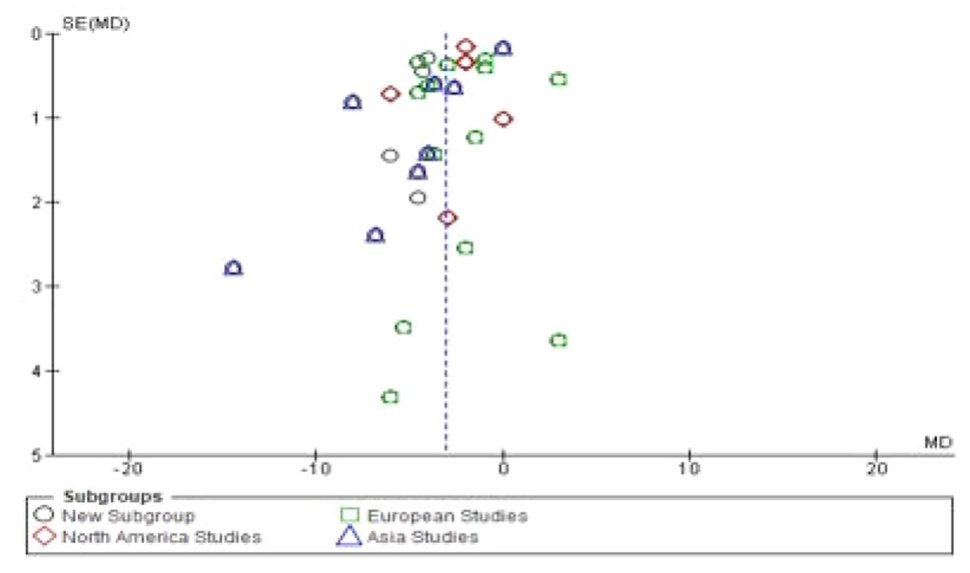
Funnel plots for length of stay

**Fig. 4:**
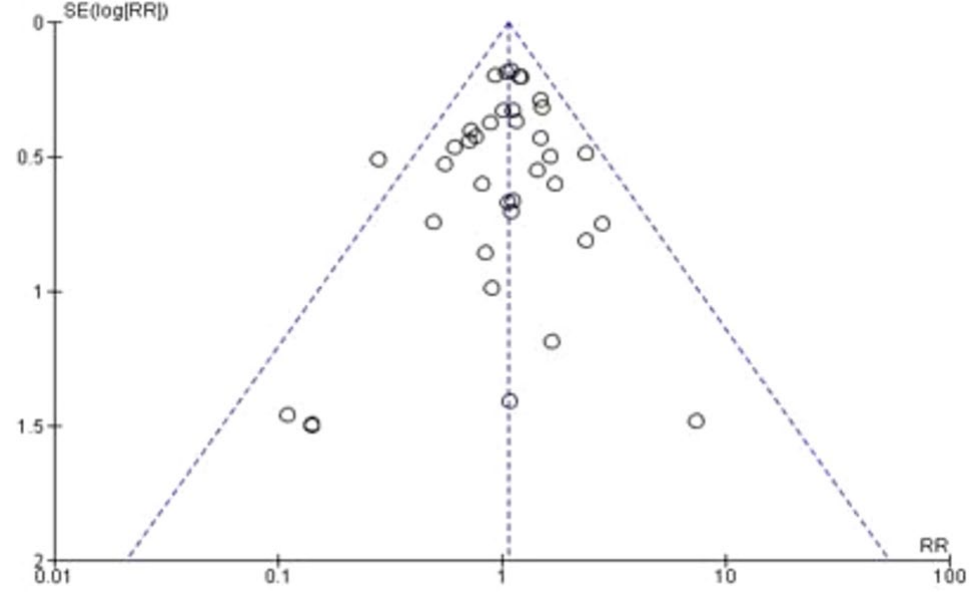
Funnel plots for readmission rates

Funnel plots for LOS and readmission rates were used to assess publication bias as shown in Figs. 3, 4. The asymmetry of the funnel plots suggested no evidence of publication bias. In the presence of heterogeneity, a sensitivity analysis was conducted to test the reliability of the results.

### Hospital costs

**Fig. 5:**
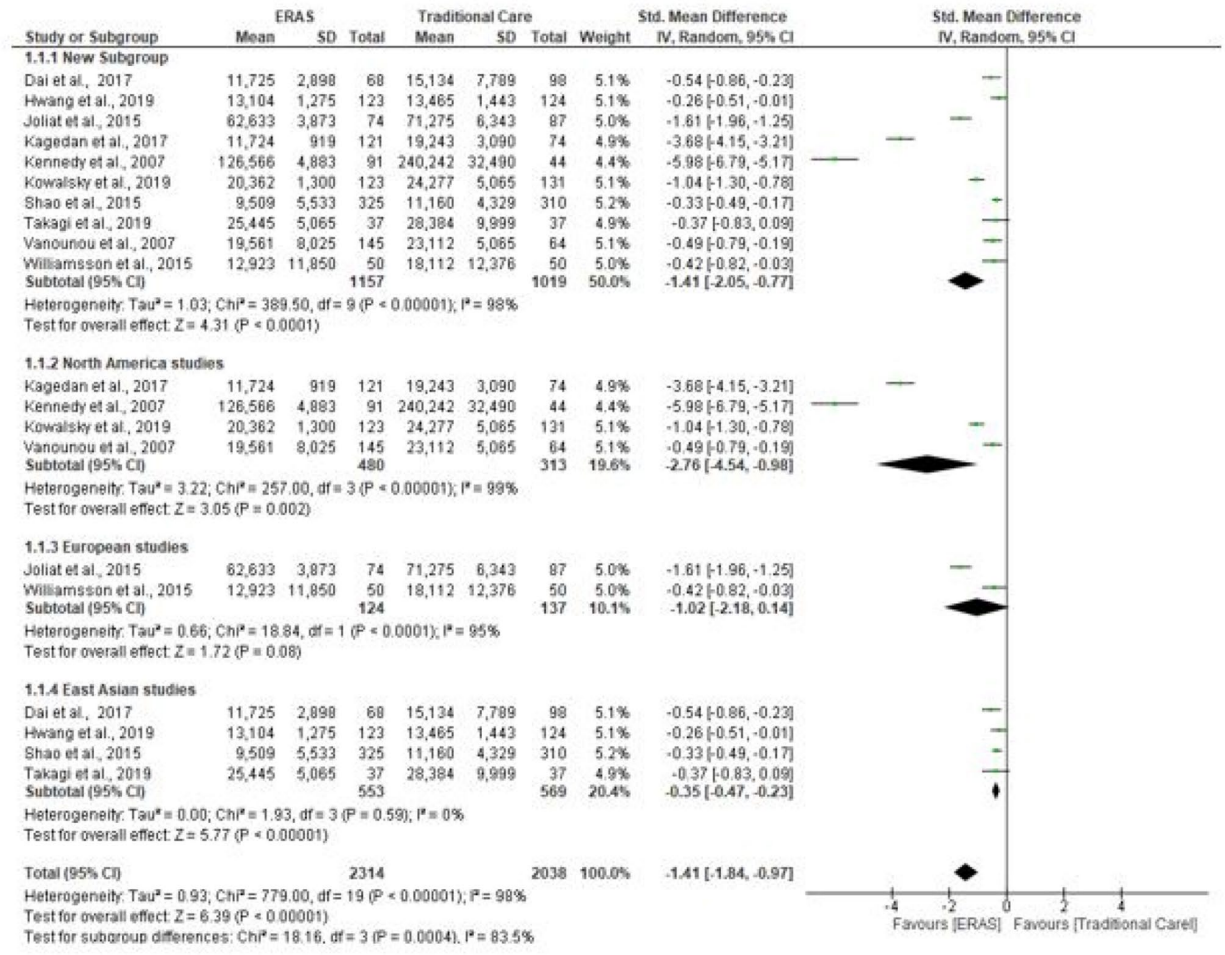
Forest plot of hospital cost, ERAS vs traditional care; subgroup analysis (North America, Europe and Asia studies)

Ten studies evaluated hospital costs (3378 patients). Four of the studies measured hospital costs in US dollar (Takagi et al. 2019; Kennedy et al 2007; Kowalsky et al. 2019; Vanounou et al. 2007), two in Chinese yuan (Shao et al. 2015; Dai et al. 2017), two in euros (Joliat et al. 2015; Williamsson et al. 2015), one each in Canadian dollar (Kagedan et al. 2017) and South Korean won (Hwang et al. 2019). The pooled analysis suggested hospital costs were significantly lower in the ERAS group compared to the traditional care group (SMD = − 1.41; CL, − 2.05 to − 0.77; *P* < 0.00001). However, there was significant evidence of heterogeneity observed in the studies (*χ*^2^ = 389.50; df = 9; *P* < 0.00001; *I*^2^ = 98%). Similarly, in the subgroup analysis of studies conducted in different continents, hospital costs were lower in the ERAS group in studies conducted in North America (SMD = − 2.76; CL, − 4.54 to − 0.98; *P* = 0.002) and East Asia (SMD = − 0.35; CL, − 0.47 to − 0.23; *P* < 0.00001), while there was no difference in studies conducted in Europe (SMD = − 1.02; CL, − 2.18–0.14); *P* = 0.08). There was evidence of substantial heterogeneity in studies conducted in North America (*χ*^2^ = 257.00; df = 3; *P* < 0.00001; *I*^2^ = 99%) and Europe (*χ*^2^ = 18.84; df = 1; *P* < 0.0001; *I*^2^ = 95%). On the contrary, there no evidence of heterogeneity in studies conducted in Asia (*χ*^2^ = 1.93; df = 3; *P* = 0.59; *I*^2^ = 0%). There was a significant difference in hospital costs across the three continents (*χ*^2^ = 18.16; df = 3; *P* = 0.0004; *I*^2^ = 83.5%). See Fig. 5.

### Length of stay

**Fig. 6:**
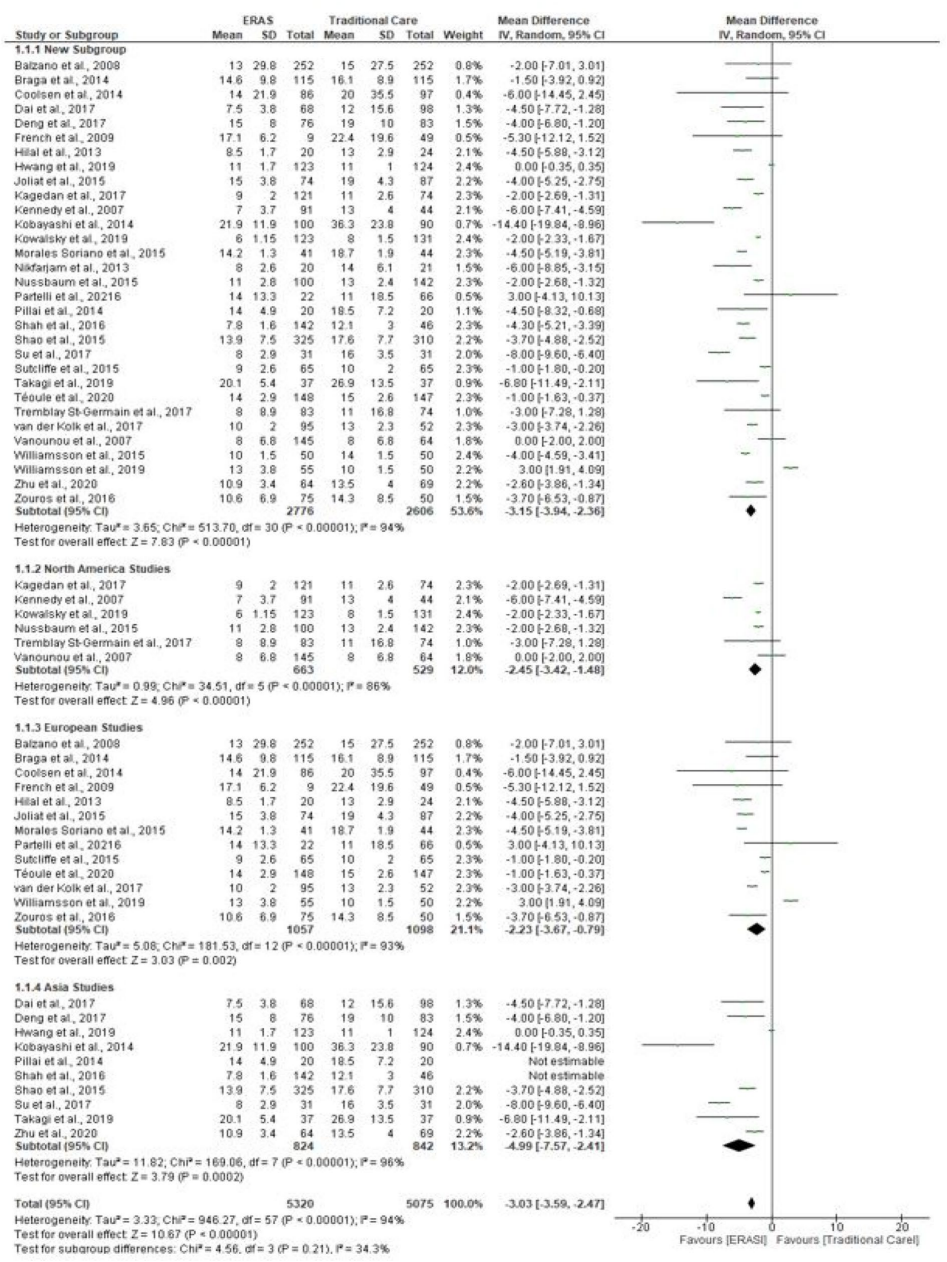
Forest plot of length of stay, ears vs traditional care; subgroup analysis (North America, Europe and Asia studies)

Length of stay was reported in all studies. Pooling of all results demonstrated a significant reduction in LOS in the ERAS group compared to the traditional care group (MD = − 3.15; CI, − 3.94 to − 2.36; *P* < 0.00001), with evidence of heterogeneity (*χ*^2^ = 513.70; df = 30; *P* < 0.00001; *I*^2^ = 94%). In addition, a subgroup analysis demonstrated a shorter LOS after implementation of ERAS in studies conducted in North America (MD = − 2.45; CI, − 3.42 to − 1.48; *P* < 0.00001), Europe (MD = − 2.23; CI, − 3.67 to − 0.79; *P* = 0.002) and Asia (MD = − 4.99; CI, − 7.57 to − 2.41; *P* = 0.0002). There was no significant difference in LOS in the three continents (*χ*^2^ = 4.56; df = 3; *P* = 0.21, *I*^2^ = 34.3%). See Fig. 6.

### Complication rates

**Fig. 7:**
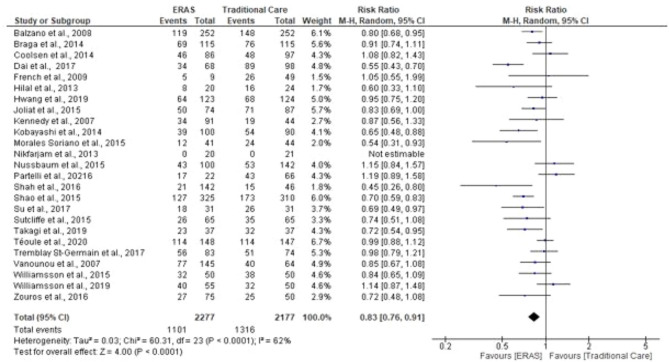
Forest plot of overall complication rates, ERAS vs traditional care

**Fig. 8:**
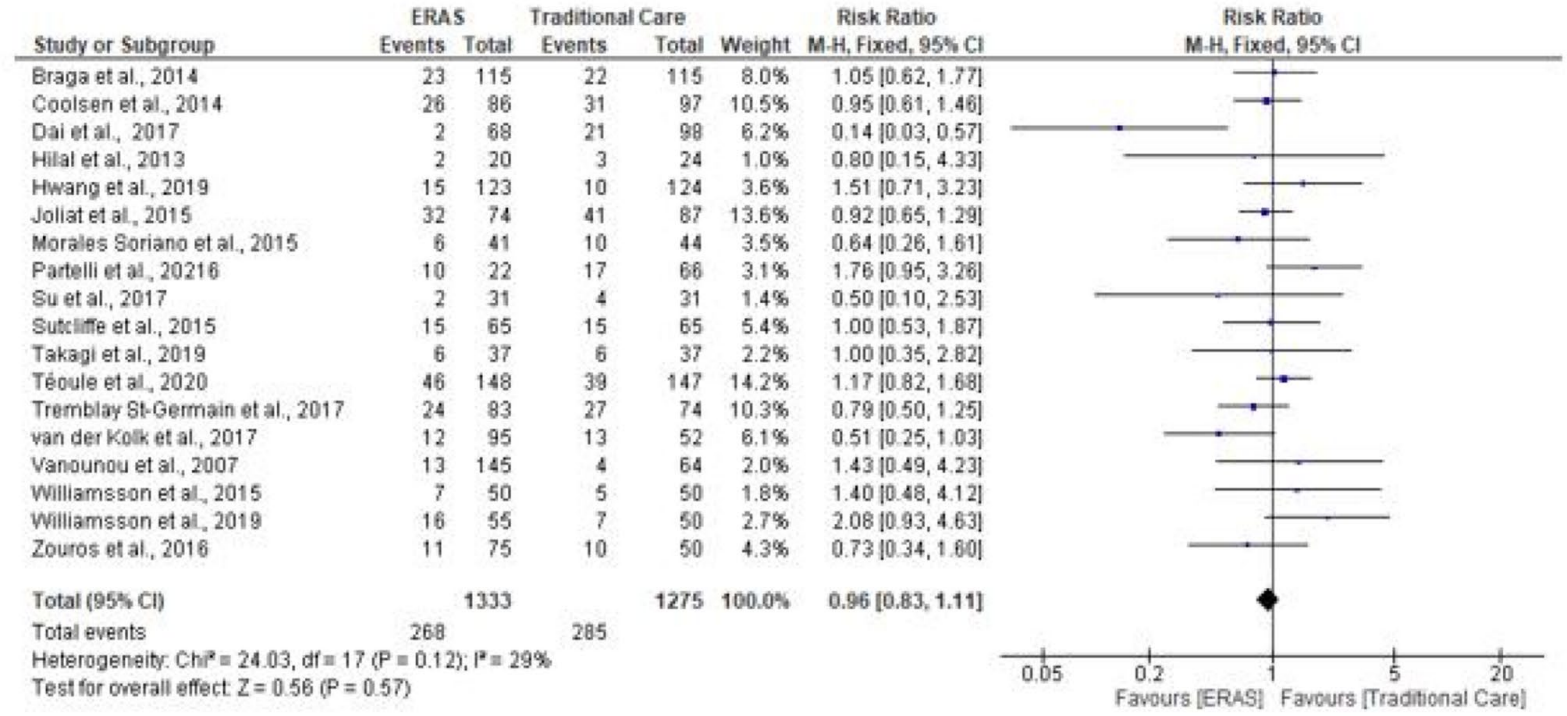
Forest plot of major complication rates, ERAS vs traditional care

Twenty-five reported incidences of complications. Overall complications were reported in thirty-four studies (4454 patients). A total of 2417 patients experienced complications, 1101 patients in ERAS groups compared to 1316 in traditional care groups. One study reported no complication in both the ERAS and traditional groups (Nikfarjam et al. 2013). The meta-analysis revealed a significant reduction in rates of complication in the ERAS group (RR = 0.83; CI, 0.76–0.91; *P* < 0.0001), however, there was evidence of substantial heterogeneity (*χ*^2^ = 60.31; df = 23; *P* < 0.0001; *I*^2^ = 62%). Eighteen studies provided data on major complications (2608 patients). 553 patients had major complications, 268 patients in ERAS vs 285 patients in traditional care. Pooling the results demonstrated that major complications were comparable in both groups (RR = 0.96; CL, 0.83–1.11; *P* = 0.57), with no significant evidence of heterogeneity (*χ*^2^ = 24.03, df = 17; *P* = 0.12; *I*^2^ = 29%). See Figs. 7, 8.

### Delayed gastric emptying (DGE)

**Fig. 9:**
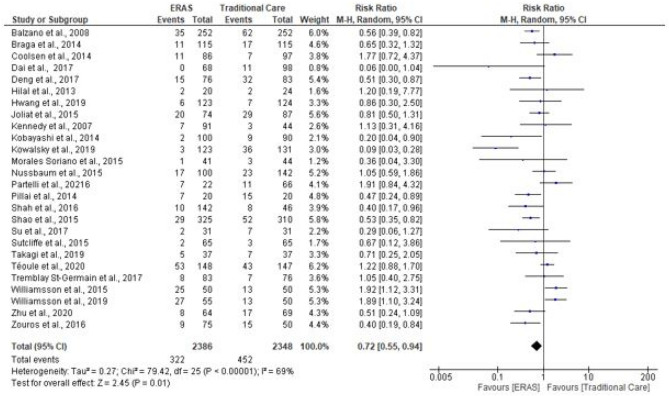
Forest plot of DGE, ERAS vs traditional care

Twenty-six studies supplied data on DGE (4734 patients). Of these, three studies recorded DGE according to their own centre definition (Kennedy et al. 2007; Su et al. 2017; Braga et al. 2014), two studies did not state how DGE was evaluated (Sutcliffe et al. 2015; Tremblay St-Germain et al. 2017), while the remaining studies defined DGE according to the International Study Group of Pancreatic Surgery (ISGPS) (Wente et al. 2007). Cases of DGE were recorded in 774 patients, with 322 being in the ERAS group compared to 452 in traditional care. The pooled analysis demonstrated significantly fewer cases of DGE in the ERAS group (RR = 0.72; CI, 0.55–0.94; *P* = 0.01). However, there was evidence of substantial heterogeneity (*χ*^2^ = 79.42; df = 25; *P* < 0.00001; *I*^2^ = 69%). See Fig. 9.

### Mortality rates

**Fig. 10:**
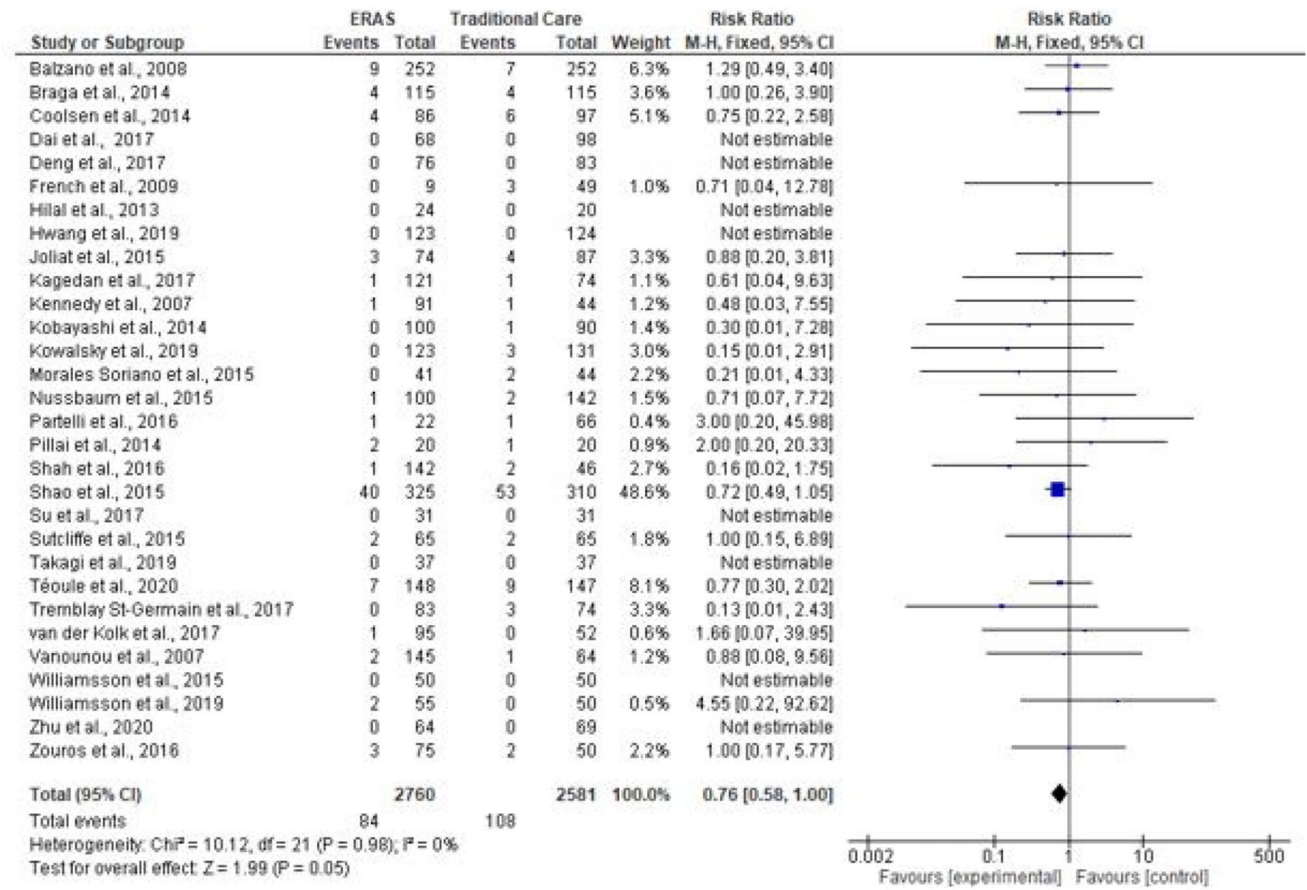
Forest plot of mortality rates, ERAS vs traditional care

Mortality rates were reported in 30 studies (5341 patients). Eight studies reported zero mortality (Deng et al. 2017), (Takagi et al. 2019; Su et al. 2017; Williamsson et al. 2015; Zhu et al. 2020; Dai et al. 2017; Hilal et al. 2013). In one study (Shao et al. 2015), mortality rates were substantially higher than normal (12% in the ERAS group vs 17.1% in the traditional care group), this was likely due to long-term follow up in the study (ranged from 1.3 to 48 months). A total of 192 deaths occurred in the studies, 84 patients in the ERAS, compared to 108 in the traditional care. On pooling the results, the number of deaths was significantly lower in the ERAS group (RR = 0.76; CI, 0.58–1.00; *P* = 0.05) and there was no evidence of heterogeneity (*χ*^2^ = 10.12; df = 21; *P* = 0.98; *I*^2^ = 0%). See Fig. 10.

### Readmission rates

**Fig. 11:**
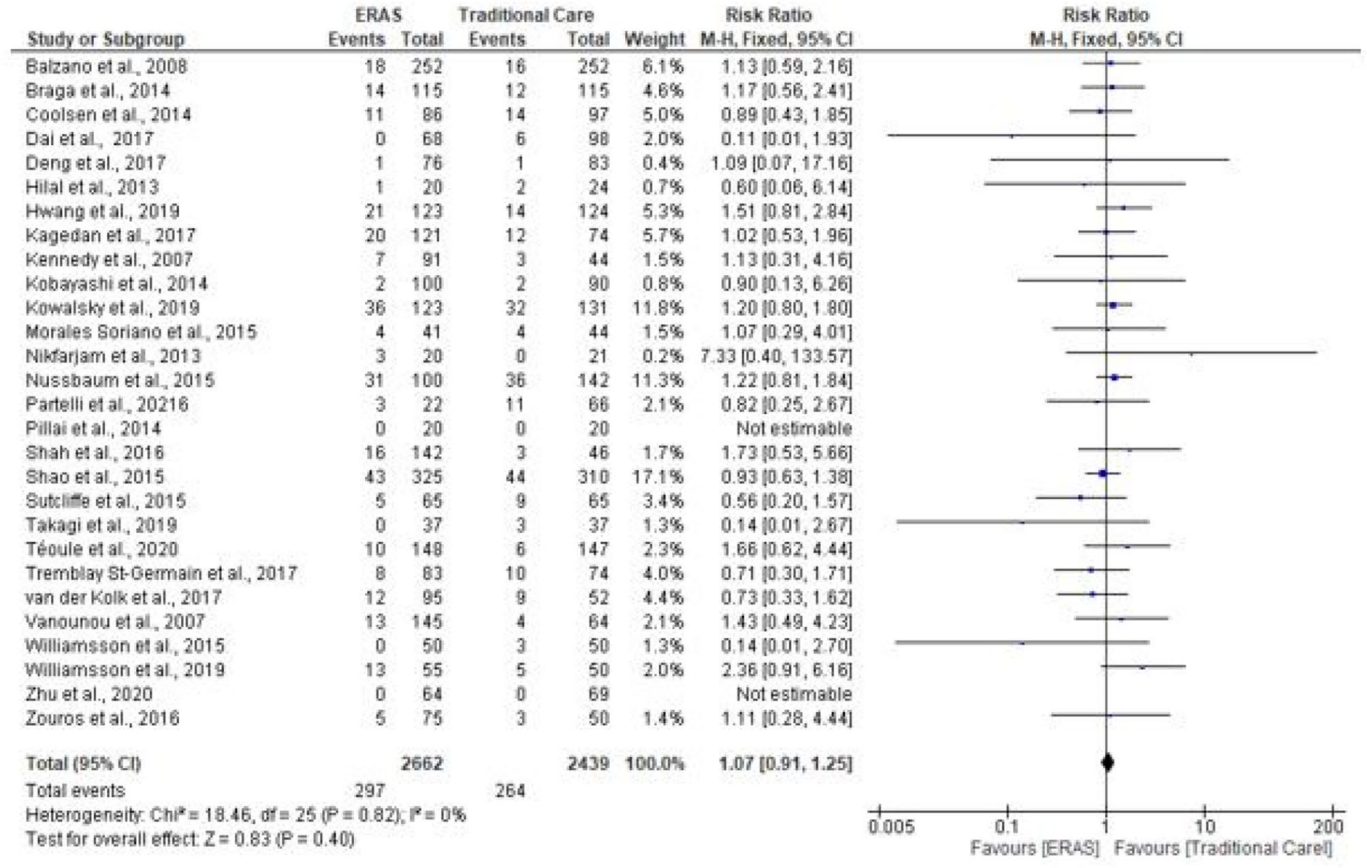
Forest plot of readmission rate, ERAS vs traditional care

Twenty-eight studies supplied data for readmissions (5101 patients). Following hospital discharge, 561 patients were readmitted within 30 days (297 in ERAS compared to 264 in traditional care). There was no difference in ERAS and traditional care after pooling the results (RR = 1.07; CI, 0.91–1.25; *P* = 0.40), with no evidence of heterogeneity observed (*χ*^2^ = 18.46; df = 25; *P* = 0.82; *I*^2^ = 0%). See Fig. 11.

### Reoperation rates

**Fig. 12:**
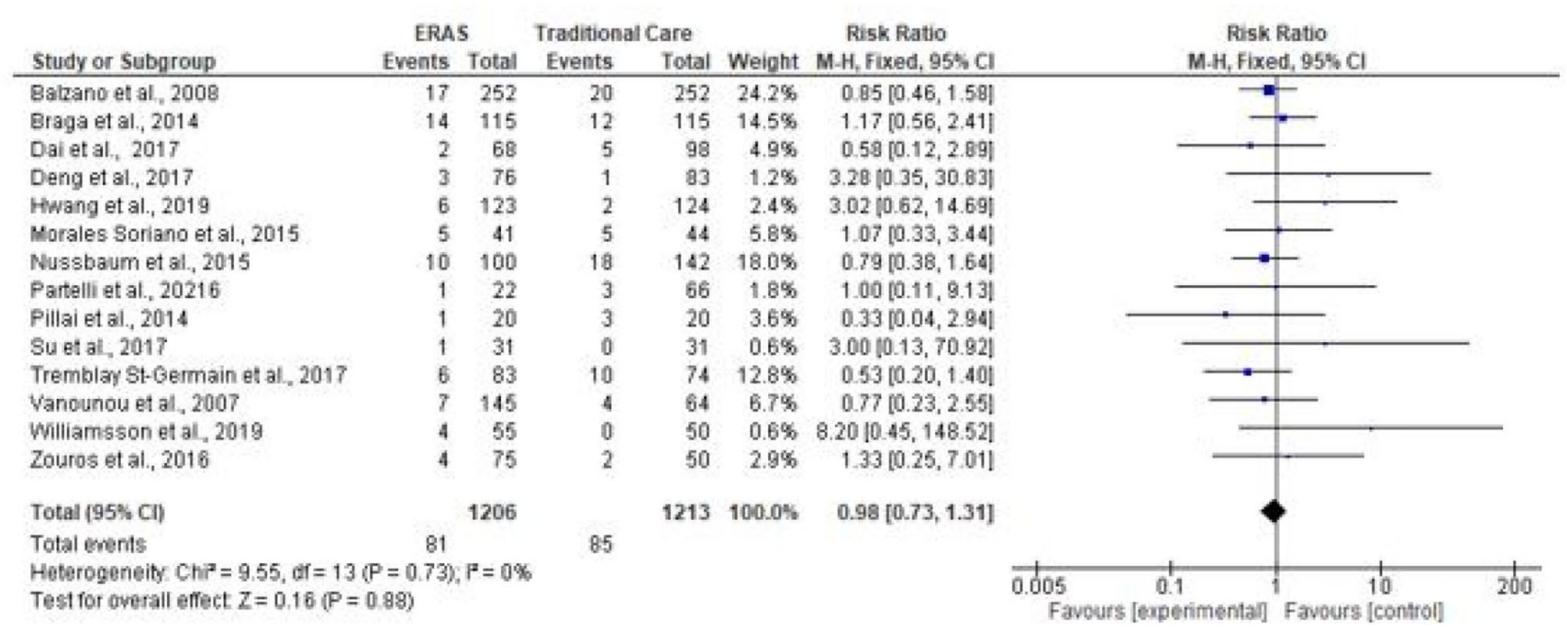
Forest plot of reoperation rates, ERAS vs traditional care

Reoperation rates were reported in fourteen studies (2419 patients). A total of 166 patients had to be reoperated, 81 patients in ERAS and 85 in traditional care. A pooled analysis found both groups to have similar reoperation rates (RR = 0.98; CI, 0.73–1.31; *P* = 0.88). There was no evidence of heterogeneity (*χ*^2^ = 9.55, df = 13; *P* = 0.73; *I*^2^ = 0%). See Fig. 12.

### Compliance

Six studies evaluated overall compliance to key elements of the ERAS pathway. Two of these studies compared rates of compliance to ERAS items between ERAS group and traditional care group. Compliance was significantly higher in ERAS group, ranging 81.2–90.3% in ERAS group compared to 34.9–43.8% in traditional care. The remaining four studies did not compare compliance between the two groups. (Joliat et al. 2015) reported 70% rates of compliance in the ERAS group, while (Van der Kolk et al. 2017) reported 80% compliance during intensive care and 60% for the surgical ward period, respectively. Similarly, Zouros et al. (Zouros et al. 2016) found compliance to 13 key ERAS items to be > 74%, with 100% compliance in five of the 13 key elements. However, (Braga et al. 2014) recorded the lowest compliance (ranged between 38 and 66%). Two studies investigated correlation between compliance and clinical outcomes. In these studies, higher compliance was associated with fewer complications (Zouros et al. 2016; Braga et al. 2014) and shorter Length of stay (Zouros et al. 2016).

## Discussion

Pancreatoduodenectomy is the most common treatment for pancreatic cancer. However, it remains one of the most complex and challenging procedures (Navarro 2017). Despite the significant improvement in outcomes such as mortality rate, complications remain as high as 60% (Lermite et al. 2013; Kunstman et al. 2019) and are the main reason for delayed discharge (Zhang et al. 2020).

This present meta-analysis included a total of 31 studies and 5382 patients making it the largest study to date on this topic. Previous systematic reviews and meta-analyses have concluded that implementation of ERAS pathways may reduce length of hospital stay and overall complications in pancreatoduodenectomy without increasing rates of mortality and readmission (Coolsen et al. 2013), (Wang et al. 2020).

With regard to the primary outcome, this review pooled sufficient data to investigate the impact of ERAS on hospital costs in pancreaticoduodenectomy. Three previous reviews included data on hospital costs in their analysis (Coolsen et al. 2013; Kagedan et al. 2015; Xiong et al. 2016), however, these data were not pooled. By contrast, this review included 10 studies on hospital costs, making it the first meta-analysis to confirm that implementation of ERAS can achieve significant cost savings in pancreatoduodenectomy. The reduction in hospital costs was also observed in the subgroup analysis of studies conducted in North America and East Asia, thereby strengthening the findings of this review. However, hospital costs varied significantly. This variation may be due to how medical costs are calculated from one centre to another. This emphasises the need for a standardised method of reporting medical costs.

Regarding secondary outcomes, this review found a significant reduction in length of stay of 3.15 days following implementation of ERAS protocols; a finding that is consistent with previous reviews on pancreatic surgery (Sun et al. 2020), (Wang et al. 2020). However, it is worth noting the presence of heterogeneity in the LOS. Despite conducting a sensitivity analysis, the heterogeneity still existed. The presence of heterogeneity could be due to several reasons, for example, how length of stay is calculated. Some studies reported LOS as either total LOS or postoperative LOS, whilst the majority of the studies did not state whether LOS was calculated as total length of stay or postoperative length of stay. Furthermore, the model of healthcare delivery differs significantly from one country to another, along with cultural ethos. For example, in countries such as the United Kingdom, it is a standard practice for a postoperative patient to be discharged from hospital to continue rehabilitation in the community. Whereas this practice is rare in many other countries and may not be affordable to patients without health insurance (Xiong, et al. 2016).This review also demonstrated that ERAS reduces cases of overall complications and delayed gastric emptying (DGE). A separate analysis was conducted to investigate the impact of ERAS on major complications. This finding was consistent with previous reviews (Bin Ji et al. 2018; Sun et al. 2020), major complications did not change in the ERAS group.

Contrary to previous reviews, mortality rates were significantly lower in the ERAS group. However, this result was swayed in favour of ERAS by a study that conducted a long-term follow-up (Shao et al. 2015). When this study was excluded from the meta-analysis, there was no significant difference between both groups (RR = 0.80; 0.55–1.17; *P* = 0.25). Therefore, the long-term impact of the ERAS pathway should be investigated further in high-quality randomised control trials (RCTs). Meanwhile, introduction of an ERAS pathway did not reduce readmissions and reoperations compared to traditional care.

The numbers of ERAS items utilised across all studies varied significantly. None of the studies included in this review applied all 27 items in the ERAS guidelines for pancreatoduodenectomy (Lassen et al. 2012; Melloul et al. 2019), with some studies using as little as six items. This is likely to be due to most studies being conducted before the first ERAS guidelines for pancreatoduodenectomy were published in 2012 (Lassen et al. 2012). The key ERAS items identified were preoperative education and counselling, minimum fasting and administration of carbohydrate drinks prior to surgery, epidural analgesia, intravenous fluids restriction, prevention of hypothermia, early removal of urinary catheters and abdominal drains, early oral intake, early mobilisation, early commencement of oral analgesia and prevention of postoperative nausea and vomiting (PONV). Early oral intake and early mobilisation were the most common interventions and were implemented in thirty-one studies, while preoperative carbohydrate drinks were the least implemented intervention and were on only administered 2–3 h prior to surgery in thirteen studies.

Most of the studies included did not investigate compliance to the ERAS pathway. When investigated, compliance rates were found to be significantly higher in the ERAS group in studies comparing compliance between the two groups (Takagi et al. 2019; Su et al. 2017). In studies that investigated compliance to key elements of ERAS in the ERAS group (Zouros et al. 2016; Braga et al. 2014), poor compliance was more prevalent in the postoperative ERAS elements particularly, oral analgesia, resumption of free fluids and normal diet and removal of abdominal drain and nasogastric tube. Moreover, patients with poor compliance experienced higher incidence of complications and prolonged hospital stay. Hence, flagging patients with poor compliance to key postoperative ERAS items may allow early identification of patients group that require additional care or further investigation.

It is worth mentioning the limitations in this current review.

1. The presence of heterogeneity was observed in hospital costs, LOS, overall complications and DGE. Where there was evidence of heterogeneity, sensitive analyses were conducted to investigate the influence of a single study by eliminating a study at each round. Despite this analysis, it was not possible to reduce the presence of heterogeneity below substantial level. Although, a random effect was used where heterogeneity could not be eliminated, however, it is not certain how this would have impacted the reliability of findings of this review.
2. None of the studies included in this review adopted current ERAS guidelines, which may have contributed to significant evidence of heterogeneity. A future study solely based on current ERAS guidelines on pancreaticoduodenectomy.
3. Most of the studies do not specify surgical approach applied in the surgeries; therefore, this review was unable to reach a conclusion on the additional benefits of minimally invasive approach in ERAS protocols. A future high quality RCTs is recommended to obtain this useful information.

## Conclusion

This current review demonstrated that the implementation of ERAS is safe and feasible in pancreaticoduodenectomy, improves clinical outcomes such as length of stay, complications, DGE and mortality rates, without changing readmission and reoperation rates, while delivering significant cost savings. High levels of compliance can be achieved in ERAS and is associated with better clinical outcomes especially LOS and complications.

Evidently, successful implementation of ERAS is dependent on compliance to key elements. Therefore, early identification of patients with poor compliance may ensure this group are given additional care to maximise clinical outcomes.
